# AntiRetroviral Therapy In Second-line: investigating Tenofovir-lamivudine-dolutegravir (ARTIST): protocol for a randomised controlled trial

**DOI:** 10.12688/wellcomeopenres.16597.1

**Published:** 2021-02-17

**Authors:** Ying Zhao, Claire Keene, Rulan Griesel, Kaneez Sayed, Zimasa Gcwabe, Amanda Jackson, Olina Ngwenya, Charlotte Schutz, Rene Goliath, Tali Cassidy, Eric Goemaere, Andrew Hill, Gary Maartens, Graeme Meintjes

**Affiliations:** 1 Department of Medicine, University of Cape Town, Cape Town, South Africa; 2 Wellcome Centre for Infectious Diseases Research in Africa, Institute of Infectious Disease and Molecular Medicine, University of Cape Town, Cape Town, South Africa; 3 Médecins Sans Frontières, Cape Town, South Africa; 4 Centre for Tropical Medicine and Global Health, Nuffield Department of Medicine, University of Oxford, Oxford, UK; 5 Division of Clinical Pharmacology, Department of Medicine, University of Cape Town, Cape Town, South Africa; 6 Division of Public Health Medicine, School of Public Health and Family Medicine, University of Cape Town, Cape Town, South Africa; 7 Department of Pharmacology and Therapeutics, University of Liverpool, Liverpool, UK

**Keywords:** Second-line, antiretroviral therapy, dolutegravir, HIV, randomised controlled trial

## Abstract

**Background:** Dolutegravir has superior efficacy and tolerability than lopinavir-ritonavir in second-line antiretroviral therapy after failure of a first-line non-nucleoside reverse transcriptase inhibitors-based regimen, when dolutegravir is accompanied by at least one fully active nucleoside reverse transcriptase inhibitor (NRTI). Resistance testing to select NRTIs is not feasible in low- and middle-income countries due to cost and limited laboratory capacity. Evidence suggests that recycling tenofovir plus lamivudine or emtricitabine backbone with dolutegravir could provide an effective second-line option. This study aims to determine the virologic efficacy of tenofovir-lamivudine-dolutegravir (TLD) with and without a lead-in supplementary dose of dolutegravir (to counteract the inducing effect of efavirenz) in patients failing a first-line regimen of tenofovir-emtricitabine-efavirenz (TEE).

**Methods:** We will perform a parallel group, randomised (1:1), double blind, placebo-controlled, Phase II trial, comparing TLD fixed dose combination daily with a lead-in supplementary 50 mg dolutegravir dose versus matching placebo taken 12 hours later for the first 14 days, in patients failing a first-line TEE regimen. The trial will be set in two primary care clinics in Khayelitsha; a large, peri-urban informal settlement in Cape Town, South Africa. We will enrol 130 participants, with follow-up to 48 weeks. The primary endpoint is proportion achieving viral load <50 copies/mL at week 24 using a modified intention-to-treat analysis and the U.S. Food and Drug Administration snapshot algorithm. Secondary endpoints include virologic suppression at weeks 12 and 48, time to suppression, emergence of dolutegravir and new NRTI resistance mutations, safety, and tolerability.

**Discussion:** Impaired viral fitness due to NRTI resistance mutations and dolutegravir’s high barrier to resistance provide rationale for switching patients from a failing TEE regimen to TLD; however, clinical evidence regarding virologic efficacy is lacking. This study provides estimates of such a strategy’s early virologic efficacy with and without a supplementary dolutegravir dosing.

**Registration: **ClinicalTrials.gov NCT03991013 (19/06/2019).

## Introduction

### Background

As of the end of June 2020, 26 million people living with HIV (PLWH) were accessing antiretroviral therapy (ART) ^1^. The ideal properties of ART include a low pill burden, good tolerability, low potential for drug-drug interaction, no requirement for laboratory safety monitoring, a high genetic barrier to resistance, and low cost. Dolutegravir, an integrase strand transfer inhibitor (InSTI), fulfils many of these criteria ^2– 5^. As second-line ART in the DAWNING study, dolutegravir with two nucleoside reverse transcriptase inhibitors (NRTIs) was superior in safety and efficacy to lopinavir-ritonavir with two NRTIs, achieving 82% virologic suppression at week 24 (defined as HIV viral load [VL] <50 copies/mL) compared to 69% of participants receiving lopinavir-ritonavir ^6^. This provides compelling evidence to consider dolutegravir as the preferential second-line option. However, there is an important caveat: at least one of the two NRTIs selected for DAWNING participants had to be fully active based on resistance testing at baseline. Resistance testing is not widely available in low- and middle-income countries (LMICs), and its use is restricted by cost to second-line failures only. Without resistance testing to determine the optimum regimen, the World Health Organization (WHO) guidelines recommend switching from a failing tenofovir-based first-line regimen containing a non-nucleoside reverse transcriptase inhibitor (NNRTI) to a second-line regimen of zidovudine-lamivudine-dolutegravir to ensure that at least one NRTI is fully active ^7^.

### Choice of NRTI backbone

Recycling tenofovir plus lamivudine or emtricitabine (XTC) in second-line regimen after virologic failure instead of switching to zidovudine is a desirable strategy. Zidovudine is less well tolerated compared with tenofovir, requires twice daily dosing, needs intensive early monitoring for haematological toxicity, and does not protect patients co-infected with hepatitis B virus ^8^. However, prevalence of tenofovir resistance after virologic failure with a first-line regimen was 57% in sub-Saharan Africa, and high-level resistance to lamivudine and emtricitabine (M184V/I mutations) was found in 83% of those with tenofovir resistance ^9^. Switching to tenofovir-lamivudine-dolutegravir (TLD) second-line by changing only one antiretroviral (i.e. replacing efavirenz with dolutegravir) risks inadvertent dolutegravir monotherapy. Dolutegravir has a high genetic barrier to resistance, but resistance has been described using dolutegravir monotherapy as maintenance in the DOMONO and MONCAY studies ^10, 11^.

It is well established that NRTI resistance impairs viral fitness in both *in vitro* and clinical studies ^12, 13^, particularly the M184V/I mutations. In the EARNEST trial, the NRTI-lopinavir-ritonavir combination outperformed lopinavir-ritonavir monotherapy in participants who were no longer responding to a first-line regimen containing two NRTIs, even though 95% of participants had at least one NRTI mutation and 59% had no fully active NRTI ^14^. Dolutegravir selects for R263K resistance mutation. Under dolutegravir drug pressure *in vitro*, only the wild-type virus was able to acquire R263K mutation, and K65R mutation (conferring intermediate resistance to tenofovir) and M184I/V mutations protected against the development of dolutegravir resistance ^15^.

### Rationale for evaluating dolutegravir supplementary dosing strategy

Efavirenz induces enzymes (UGT1A1 and CYP3A4) and transporters (P-glycoprotein and breast cancer resistance protein) involved with dolutegravir absorption and metabolism, which reduces plasma dolutegravir concentrations at the end of the dosing interval up to 75% ^16, 17^. However, simulations of switching from efavirenz- to dolutegravir-based regimens estimated that efavirenz concentrations remain above the minimum effective concentration (MEC) up to three days after discontinuation, and dolutegravir trough concentrations reach MEC three days after switch ^16^. Efavirenz plasma concentrations are greatly influenced by *CYP2B6* metabolizer genotypes as efavirenz is primarily metabolised by CYP2B6 ^18^. The prevalence of slow metabolizer *CYP2B6* genotype in sub-Saharan African population is 15–20% ^19^. In slow metabolizers, dolutegravir trough concentrations reach MEC six days post switch and efavirenz concentrations take eight days to drop below MEC ^16^.

Generaux *et al.* thus predicted that no dolutegravir dose adjustment is required when switching from an efavirenz-based regimen in virologically suppressed patients ^16^. A pharmacokinetic sub-study of the STRIIVING study confirmed this, finding that dolutegravir plasma mean concentrations were maintained above the concentrations required for 90% inhibition from one week after switching from an efavirenz-based regimen ^20^. However, in patients switching with a raised VL and in the presence of efavirenz and NRTI resistance mutations, exposure to sub-therapeutic plasma trough concentrations of dolutegravir in the immediate post-switch period could drive the development of dolutegravir resistance mutations; hence there may be a requirement for increased dosing of dolutegravir during the initial period of switching to TLD to overcome the residual efavirenz inducing effect.

We conducted a single arm, prospective, interventional study (ClinicalTrials.gov NCT03991013 [19/06/2019]) of patients switching to TLD after failing a tenofovir-based first-line regimen containing efavirenz or nevirapine (Keene C, Griesel R, Zhao Y, *et al.* Virologic efficacy of tenofovir, lamivudine and dolutegravir as a second-line regimen in adults who have failed a tenofovir-containing first-line regimen: a prospective cohort study. 2020. unpublished report). The fixed dose combination of TLD was supplemented with a lead-in 50 mg dose of dolutegravir taken 12 hours later for the first 14 days to compensate for the inducing effect of efavirenz on dolutegravir metabolism and transport that persists for around two weeks after efavirenz is stopped. A high proportion of virologic suppression (51/60 [85%, 95% confidence interval 73 - 93%]) was achieved at week 24 despite substantial baseline NRTI resistance, suggesting that when dolutegravir is used with tenofovir and lamivudine, even if both NRTIs are compromised by resistance mutations, the residual activity and crippling will result in an effective second-line regimen. No dolutegravir resistance mutations was observed in the one participant that met the protocol-defined criteria for resistance testing.

## Study hypotheses

We hypothesise that a lead-in supplementary dose of dolutegravir will reduce the risk for treatment failure and the development of dolutegravir resistance in patients initiated on a second-line regimen of TLD. The alternative hypothesis is that virologic suppression and protection from the emergence of dolutegravir resistance will be comparable with and without a lead-in dolutegravir dose.

## Study aim

The aim of this study is to determine the proportion of patients achieving virologic suppression when recycling the tenofovir plus XTC backbone with dolutegravir (TLD combination) as a second-line regimen with and without a lead-in supplementary dose of dolutegravir, in patients failing a first-line regimen of tenofovir-emtricitabine-efavirenz (TEE).

## Study objectives

### Primary objective

To describe the proportion of patients achieving HIV VL <50 copies/mL at week 24 on TLD with and without a lead-in supplementary dose of dolutegravir, as a second-line regimen in patients who have failed a TEE first-line regimen.

i. Overall.
ii. Stratified by the presence or absence of resistance to both tenofovir and XTC on initiation of the TLD second-line regimen.

### Secondary objectives

1) To describe the proportion of patients achieving HIV VL <50 copies/mL at weeks 12 and 48 on TLD with and without a lead-in supplementary dose of dolutegravir, as a second-line regimen in patients who have failed a TEE first-line regimen.

i. Overall.
ii. Stratified by the presence or absence of resistance to both tenofovir and XTC on initiation of the TLD second-line regimen.

2) To determine the proportion of patients developing dolutegravir and new NRTI resistance mutations on the TLD second-line regimen.

3) To determine whether the development of dolutegravir resistance is associated with the presence or absence of resistance to both tenofovir and XTC on initiation of the TLD second-line regimen.

4) To describe the resistance profile of patients failing a TEE first-line regimen in this setting.

5) To evaluate dolutegravir trough concentrations when switching from an efavirenz-based first-line regimen, and to assess the pharmacokinetic requirement for a lead-in supplementary dose of dolutegravir.

6) To describe markers of adherence between those failing TLD at weeks 24 and 48, and to compare to a group of matched controls.

7) To describe other clinical characteristics, adverse events, and all-cause mortality among the study cohort.

## Ancillary studies

Several ancillary studies will be conducted within the trial. These include:

### Clinical pharmacology study

A pharmacokinetic sub-study will be conducted in 24 participants (the first 24 participants who consent and are logistically feasible for enrolment into the pharmacokinetic sub-study), to assess the trough concentrations of dolutegravir and off-treatment concentrations of efavirenz at days 3, 7, 14, and 28. Eligibility criteria are not different from eligibility for the parent study, with the only additional inclusion criterion being willingness to participate.

### Genetic sub-study

All consenting participants will have an extra blood sample taken at enrolment for DNA and stored for genetic testing at the end of the study, to determine efavirenz metabolizer genotypes. We will also store DNA for future genetic studies that are related to HIV and its treatment, such as evaluation for genetic polymorphisms associated with dolutegravir metabolism. A separate informed consent for genetic testing will be performed at the screening visit.

## Study design

**Figure 1.**
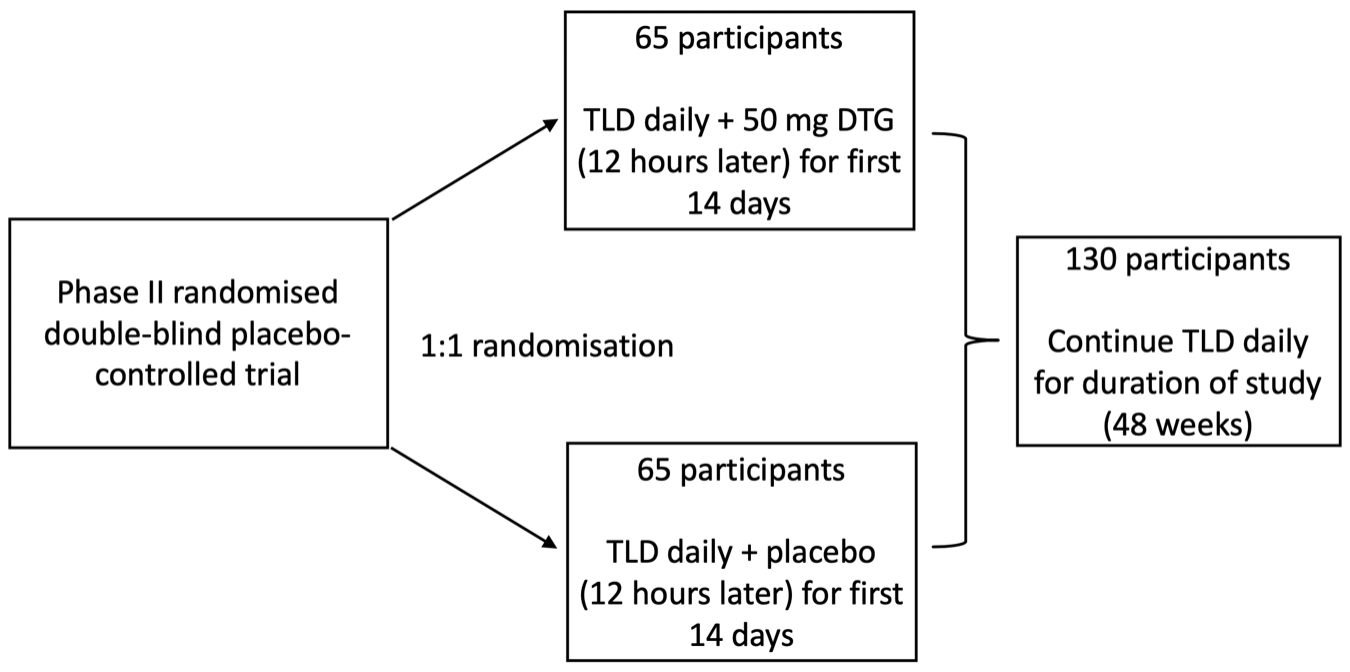
Schematic of study design. Abbreviations: TLD = tenofovir-lamivudine-dolutegravir fixed dose combination; DTG = dolutegravir.

This study will be a Phase II, randomised, double-blind, placebo-controlled trial of TLD fixed dose combination daily with a lead-in supplementary 50 mg dose of dolutegravir versus matching placebo taken 12 hours later for the first 14 days, in patients failing a first-line TEE regimen. We will describe virologic suppression in two parallel arms ( Figure 1), but will not be powered for a formal non-inferiority comparison between the two:

**Arm 1**: patients initiated on dolutegravir at 50 mg once a day as part of a fixed dose combination of TLD, with a lead-in supplementary 50 mg dose of dolutegravir taken 12 hours later for the first 14 days, with continuation of TLD for the duration of the study (48 weeks).

**Arm 2**: patients initiated on dolutegravir at 50 mg once a day as part of a fixed dose combination of TLD, with a matching placebo taken 12 hours later for the first 14 days, with continuation of TLD for the duration of the study (48 weeks).

## Study setting

The study will be conducted in the ART clinics at Michael Mapongwana Community Health Centre (CHC) and Ubuntu Antiretroviral Clinic at Site B CHC in Khayelitsha. Khayelitsha is a large, peri-urban informal settlement in Cape Town, South Africa. It is home to approximately 500,000 people, most of whom speak isiXhosa as their first language. It has a large population of PLWH.

## Endpoints

### Primary endpoint

Proportion (with 95% confidence interval [CI]) with HIV VL <50 copies/mL at week 24 using a modified intention-to-treat (mITT) analysis and according to the U.S. Food and Drug Administration (FDA) snapshot algorithm.

i. Overall.
ii. Stratified by the presence or absence of resistance to both tenofovir and XTC on initiation of the TLD second-line regimen.

### Secondary endpoints

1) Virologic suppression

a) Proportions (with 95% CI) with HIV VL <50 copies/mL at weeks 12 and 48 using a mITT analysis and according to the FDA snapshot algorithm.
  i. Overall.
  ii. Stratified by the presence or absence of resistance to both tenofovir and XTC on initiation of the TLD second-line regimen.
b) Proportions (with 95% CI) with HIV VL <50 copies/mL at weeks 12, 24, and 48 using the as treated analysis.
c) Time to virologic suppression.

2) Resistance

a) Resistance profile at baseline (NRTI and efavirenz resistance).
b) Emergence of dolutegravir and new NRTI resistance mutations in those who experience virologic failure on the TLD second-line regimen (defined as having two consecutive VLs >1000 copies/mL after week 12).

3) Safety and tolerability

a) Clinical grade 3 or 4 adverse events (AEs) as classified by the Division of AIDS Grading Table, laboratory grade 3 or 4 AEs, serious AEs, and AEs requiring discontinuation of any antiretroviral drug in the TLD second-line regimen.
b) 12-month mortality (all-cause).
c) Sleep assessment measured with the Insomnia Severity Index at baseline and at every visit.
d) Neuropsychiatric assessment measured with a test battery at weeks 0, 2, 4, 12, 24, and 48. The test battery includes the Brief Symptoms Inventory – 18, Anxiety Subscale and the Centre for Epidemiologic Studies Depression Scale.
e) Neurocognitive performance measured with the Simioni Neurocognitive Symptom Questions and the Cognitive Assessment Tool – Rapid Version 2.0 at weeks 0, 2, 4, 12, 24, and 48.

4) CD4 counts at weeks 24 and 48 and change in CD4 counts from week 0.

5) Adherence to ART, as indicated by tenofovir diphosphate concentrations at weeks 0, 12, 24, and 48, in those who are not virologically suppressed, and in matched controls from among those who are suppressed at weeks 24 and 48.

### Tertiary endpoints

Pharmacokinetic parameters and genetic polymorphisms related to the ancillary studies will be reported separately from the main trial.

## Inclusion and exclusion criteria

### Inclusion criteria

HIV positive patients ≥18 years old, who have failed their first-line TEE regimen, are able to attend the study clinic for one year of scheduled visits and who have given written, informed consent. Virologic failure is defined as having a VL of >1000 copies/mL (within the previous two months) and an immediately prior VL >1000 copies/mL, taken 2–24 months prior. In female patients of child-bearing potential, those willing to use effective and reliable contraception for the duration of the study will be eligible.

### Exclusion criteria

>2 log drop in VLs between the most recent VL and the immediately prior VL (taken 2–3 months prior); CD4 count <100 cells/μl; estimated glomerular filtration rate (eGFR) <50 ml/min/1.73m ^2^ using the Modification of Diet in Renal Disease (MDRD) formula; alanine aminotransferase >100 U/L; total bilirubin >twice the upper limit of normal; pregnant or breastfeeding; on treatment for active tuberculosis or concern that patient has undiagnosed active tuberculosis; active malignancy; allergy or intolerance to one of the antiretroviral drugs in the TLD regimen; current psychiatric disease or substance abuse judged likely to impact adherence; on treatment for AIDS-defining condition; or any other clinical condition that in the opinion of an investigator puts the patient at increased risk if participating in the study.

## Recruitment and randomisation

### Recruitment

**Figure 2.**
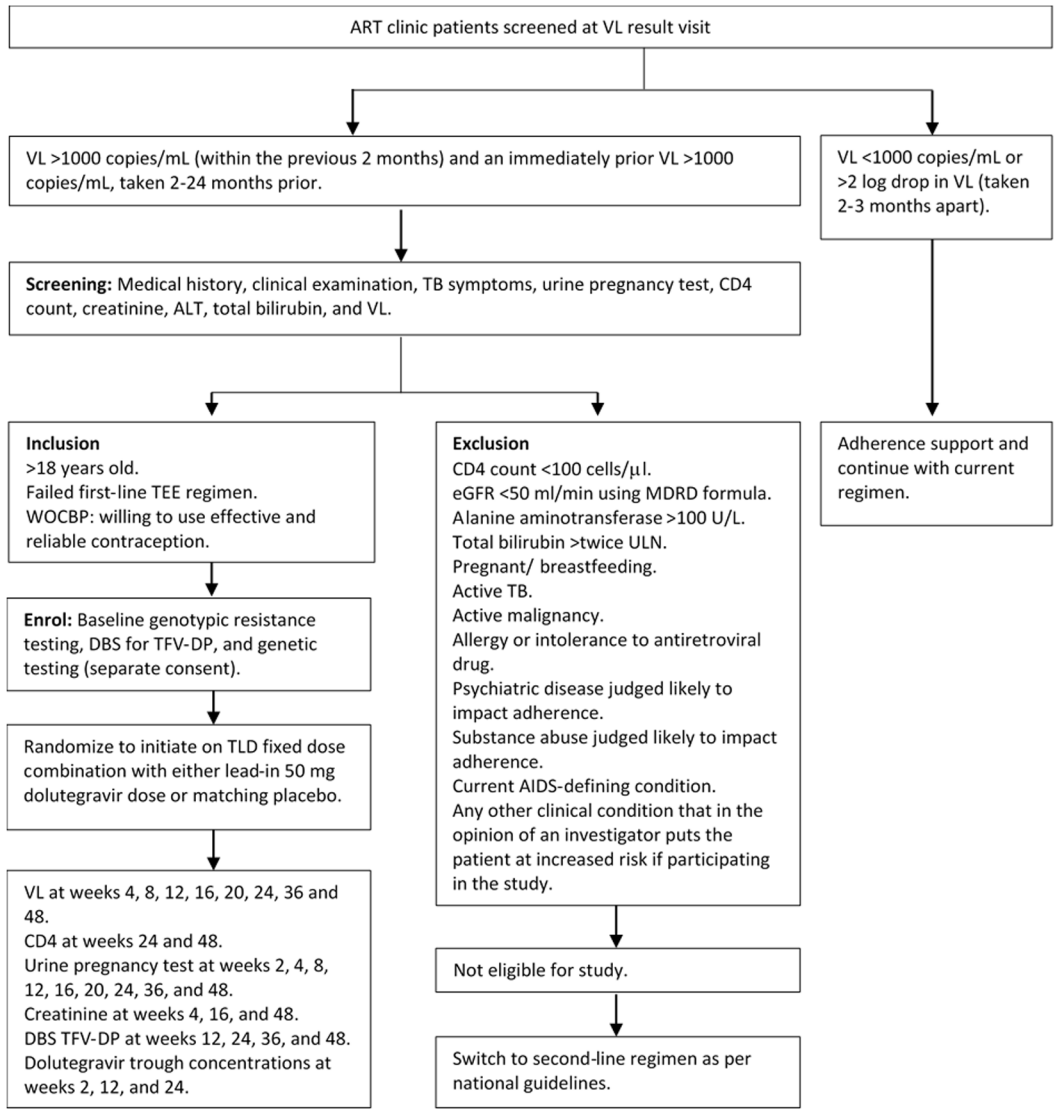
Trial schema. Abbreviations: TLD = tenofovir-lamivudine-dolutegravir; TEE = tenofovir-emtricitabine-efavirenz; ART = antiretroviral therapy, VL = viral load; TB = tuberculosis; eGFR = estimated glomerular filtration rate; MDRD = Modification of Diet in Renal Disease; DBS = dried blood spots; TFV-DP = tenofovir diphosphate; ULN = upper limit of normal; WOCBP = women of child-bearing potential.

Patients will be recruited from the ART clinics at study sites ( Figure 2). The clinical service clinicians will identify eligible patients from their routine VLs, explain the purpose of the study, and make clear that the patients’ routine care is in no way contingent on participation in the study. If the patient agrees, they will be referred to the study for screening and enrolment. The patient will be contacted by the study staff. They will be taken through the process of informed consent and will have the study explained in detail by the counsellor, with the study nurse and doctor available to answer any questions. If they agree, informed consent will be taken.

### Randomisation

Participants will be randomized in a 1:1 ratio to receive TLD fixed dose combination daily with either a lead-in supplementary 50 mg dose of dolutegravir or matching placebo. Randomization will take place on the same day that study medications are to be started (i.e. the enrolment visit). A randomisation sequence will be prepared by an Independent Pharmacist before the trial commences using block randomisation utilizing a block size of 10. The Independent Pharmacist will prepare opaque sealed envelopes (to ensure allocation concealment) labelled 1 to 130 containing the allocation assignment for the sequentially enrolled participants. The Independent Pharmacist will share the randomisation sequence with the Study Pharmacist and Study Statistician. Only the Independent and Study Pharmacists, and Study Statistician will have access to the randomisation code. All participants and investigators will be blinded to treatment allocation.

## Blinding and emergency unblinding

### Blinding

The Study Pharmacist will re-package dolutegravir and placebo tablets into identical screw-cap polypropylene containers. The study drug containers will be identical except for a label that contains the randomisation treatment number. A delegated study staff member will collect the study medication and not open the containers with either dolutegravir or placebo when handing medication to participants, to ensure that investigators remain blinded to the allocation of study medication. All documents that could potentially unblind the participant’s assignment will be kept in locked cupboards within the study pharmacy.

### Unblinding

Unblinding of the randomization allocation would only occur under exceptional circumstances after a decision is taken to stop the study medication, and when this information is deemed essential for ongoing clinical management by an attending clinician. This would usually be in the scenario where the participant has deteriorated clinically, and it is necessary to establish if the participant is receiving additional dolutegravir or placebo to direct further investigations or treatment. If the decision to unblind is taken after discussion with the Principal Investigator, that participant’s randomization allocation will be obtained from the Study Pharmacist without obtaining the randomization allocations of any of the other participants.

## Trial management

### Intervention

At the enrolment visit, participants will receive the first two-week supply of TLD fixed dose combination, and either 14 tablets of dolutegravir 50 mg or 14 tablets of placebo to take 12 hours after taking TLD. Participants will receive a two-week supply of TLD at the week 2 visit, then a four-week supply of TLD at each subsequent study visit until the end of their follow-up period (48 weeks). TLD fixed dose combination consists of tenofovir 300 mg, lamivudine 300 mg, and dolutegravir 50 mg.

### Prophylaxis of opportunistic infections

Participants with CD4 count <200 cells/mm ^3^ will receive co-trimoxazole 160/800 mg daily according to national guidelines. If the participant develops mild or moderate hypersensitivity to co-trimoxazole, it can be substituted with dapsone 100 mg daily. Participants will initiate isoniazid preventive therapy (IPT) at enrolment visit unless they have previously received IPT for at least six months or have known contraindications. IPT consists of isoniazid 5 mg/kg/day (maximum 300 mg) and pyridoxine 25 mg daily for 12 months. 

### Use of interacting medications

Polyvalent cations (calcium, magnesium, iron, and aluminium) bind to dolutegravir and reduce its absorption. Participants taking polyvalent cations will be instructed to take study medications either two hours before or six hours after. Dolutegravir inhibits an efflux transporter important in the elimination of metformin. Maximum metformin dose is 500 mg twice daily when taken with dolutegravir.

### Management of suspected tuberculosis

Active tuberculosis and concern that patient has undiagnosed tuberculosis are exclusion criteria if present at screening or enrolment visit. If a participant develops symptoms suggestive of tuberculosis they will be investigated as per national guidelines. If tuberculosis is diagnosed and the participant is started on a rifampicin-containing regimen, the participant will receive an additional dose of dolutegravir to make the dose 50 mg twice daily for the duration of the anti-tuberculosis treatment and for two weeks after anti-tuberculosis treatment completion.

### Viral load monitoring

HIV VL will be performed at baseline (i.e. screening visit), and at weeks 4, 8, 12, 16, 20, 24, 36, and 48. If any VL after week 12 is >50 copies/mL, or if there is <1 log decline in VL from baseline value at any visit from week 4 onward, or if VL is suppressed at any time and subsequently rebounds to >50 copies/mL, enhanced adherence support will be given and VL repeated two weeks after enhanced adherence counselling (EAC). If there is no decline and the repeat VL is >500 copies/mL, genotypic resistance testing will be performed on a new as well as the baseline stored sample, and the case reviewed by the Trial Steering Committee to decide on further management. If the repeat VL is between 50–500 copies/mL, EAC and routine VL monitoring will be continued.

### Renal impairment

Creatinine and eGFR will be monitored at weeks 4, 16, and 48. A small increase in serum creatinine (usually 10–20 µmol/L) can be observed with dolutegravir. It relates to dolutegravir affecting tubular creatinine secretion and does not represent a decline in renal function. If eGFR declines to <50 ml/min/1.73m ^2^, tenofovir will be discontinued and replaced with zidovudine as per national guidelines. Tenofovir may be reintroduced if there is an alternative explanation for the decline in renal function once eGFR has increased to >60 mL/min/1.73m ^2^.

### Management of women of child-bearing potential

Women of child-bearing potential consenting to take part in the study will be required to commit to the use of effective and reliable contraception, including intra-uterine contraceptive device, injectable, implantable, or oral hormonal contraception, for the duration of the study (48 weeks). This is because of an identified signal indicating a potential increased risk of neural tube defects in infants born to women taking dolutegravir at conception ^21^. A pregnancy test will be done at baseline and at every study visit.

Updated results from the Tsepamo study showed declining occurrence of neural tube defects in infants born to mothers receiving dolutegravir at conception, from an estimate of 1/100 exposure after the first report in May 2018 ^21^, to 2/1000 in April 2020 ^22^. Women who fall pregnant in the 48 weeks of the trial or express intention to conceive during the study will be counselled about the potential small risk associated with the use of dolutegravir at conception to make an informed decision of whether to stay on dolutegravir or switch to protease inhibitor-based regimen. The participant will be withdrawn from the trial, but we will continue to follow-up the participant observationally. Any pregnancy complications or foetal anomalies will be reported to the University of Cape Town Human Research Ethics Committee and the Data and Safety Monitoring Committee.

## Data collection

The trial assessment schedule for ARTIST is outlined in Table 1.

**Table 1.** ARTIST trial assessment schedule. Abbreviations: DTG = dolutegravir; TFV-DP DBS = tenofovir diphosphate dried blood spots; PK = pharmacokinetics.

Visit	Screening	Week										
		0	2	4	8	12	16	20	24	36	48	52
Informed consent	X											
Eligibility assessment	X	X										
Clinical assessment	X	X	X	X	X	X	X	X	X	X	X	X
Confirm contraceptive use	X	X	X	X	X	X	X	X	X	X	X	X
Adverse event screening		X	X	X	X	X	X	X	X	X	X	X
Sleep assessment		X	X	X	X	X	X	X	X	X	X	
Neuropsychiatric and neurocognitive testing		X	X	X		X			X		X	
Urine pregnancy test	X	X	X	X	X	X	X	X	X	X	X	X
Creatinine	X			X			X				X	
alanine aminotransferase	X											
Total bilirubin	X											
HIV viral load	X			X	X	X	X	X	X	X	X	
CD4 count	X								X		X	
Storage for later DNA extraction		X										
DTG trough and efavirenz concentrations (PK sub-study)		Day 0, 3, 7	X	X								
DTG trough concentrations			X			X			X			
TFV-DP DBS		X				X			X	X	X	

### Baseline assessment

Neuropsychiatric testing, neurocognitive testing, and sleep assessment will be performed by a trained doctor or nurse at baseline (enrolment visit). Baseline genotypic resistance testing will be taken prior to the use of any study medication. Dried blood spots (DBS) will be taken for tenofovir diphosphate (TFV-DP) concentrations. An extra blood sample will be taken for genetic testing. DNA extraction will be performed at the Wellcome Centre for Infectious Disease Research in Africa laboratory at the University of Cape Town. Specimens for baseline genotypic resistance testing, TFV-DP concentrations, and genetic testing will be stored at -80°C at the Wellcome Centre for Infectious Disease Research in Africa laboratory at the University of Cape Town and analysed retrospectively at the end of the study. Stored DNA will be genotyped with the Illumina Infinium Multi-Ethnic Global BeadChip (MEGAEX), at Vanderbilt Technologies for Advanced Genomics in Nashville, Tennessee, USA.

### Follow-up assessments

Patients will be followed up at weeks 2, 4, 8, 12, 16, 20, 24, 36, 48, and 52 after enrolment. Sleep assessment will be done at every visit. Neuropsychiatric and neurocognitive testing will be done at weeks 2, 4, 12, 24, and 48. Participants will have a repeat CD4 count at weeks 24 and 48, and a repeat creatinine at weeks 4, 16, and 48. DBS will be taken for TFV-DP concentrations at weeks 12, 24, 36, and 48. Dolutegravir trough concentrations will be taken at weeks 2, 12, and 24. Specimens for TFV-DP concentrations and dolutegravir trough concentrations will be transported to the laboratory for storage and batched for analysis at the end of the study.

### Data handling and data management

Source documentation includes original notes and logs kept by research staff in the participant study files, original prescriptions, all results from the laboratory, and results from other relevant tests. Source documentation will be completed in real time when participants are assessed by study staff members. They will be reviewed regularly by another delegated staff member, to check that data are entered correctly, all data are valid, and that correct procedures are followed. The electronic Case Report Forms will be completed by data capturers on the REDCap electronic database. Two data capturers will enter the data independently. The study’s REDCap database is hosted on the University of Cape Town server. The electronic database will automatically flag discrepant data entry and incomplete, invalid, or missing data. Errors flagged will be resolved between the data capturers and corrected on the electronic database. Once enrolment and follow up are completed the Principal Investigator will have access to the final trial dataset.

### Quality control and assurance

During the study, external site monitoring will be conducted regularly to ensure that study procedures follow the International Committee of Harmonisation Good Clinical Practice (ICH-GCP) principles, and that the trial is conducted in compliance with ethics and regulatory guidelines. In addition, the study site may be subject to review by the University of Cape Town and regulatory authorities.

## Adverse events and safety reporting

### Adverse events

Specific process for identifying, documenting, and reporting AEs and Adverse Drug Reactions (ADRs) will be followed. The definitions of the EU Directive 2001/20/EC Article 2 based on the principles of ICH GCP apply to this trial protocol. The investigational medicinal products (IMPs) in this trial have been approved for use by the South African Health Products Regulatory Authority (SAHPRA) and thus we will adhere to SAHPRA guidelines for the reporting of ADRs associated with medicines which arise during clinical trials. All new clinical events will be classified by the Division of AIDS Table for Grading the Severity of Adult and Paediatric Adverse Events. In the case of a new infection (e.g. herpes zoster), the most significant clinical symptoms (e.g. rash and pain) will be listed as an AE and the clinical diagnosis will also be reported as an AE. All laboratory results will be captured into the electronic database and a report generated of grade 3 and 4 laboratory AEs. A serious adverse event (SAE) is defined as an AE that results in death, a life-threatening incident, hospitalisation, disability, congenital abnormality, or requires medical or surgical intervention to prevent permanent impairment or damage. Causality of each AE (clinical and laboratory) in relation to the IMP (TLD fixed dose combination) will be assessed based on temporal relationship and clinical judgement. There are five categories: unrelated, unlikely, possibly, probably, and definitely related.

### Safety reporting

All grade 3–5 AEs and SAEs will be documented and reported to the Data and Safety Monitoring Committee (DSMC) every two months and the University of Cape Town Human Research Ethics Committee annually. All unexpected AEs and SAEs judged to be related or possibly related to the IMP will be reported to the DSMC and ethics committee within seven days. Follow up reports will be submitted within seven days if the condition significantly worsens, results in death, or if there is a major change in diagnosis.

## Statistical analysis

### Sample size justification

Assuming virologic suppression (VL <50 copies/mL) of 82%, as achieved by the dolutegravir arm at week 24 in the DAWNING trial ^6^, is achieved using mITT analysis at week 24, a sample size of 57 will produce a 95% CI of 72 - 92%. To account for participants discontinuing the regimen or dropping out (e.g. stopping contraception), 65 patients will be enrolled into each arm (total of 130 participants).

### Primary endpoint analysis

We will determine the proportion achieving virologic suppression at week 24 in each study arm, using mITT analysis according to the FDA snapshot algorithm. The FDA snapshot algorithm regards those with VL ≥50 copies/mL, those with missing VL within the visit window, intolerance or AE due to any antiretroviral drug in the regimen requiring switch, and those with drug substitution not permitted by the protocol as failures. Loss to follow-up (LTFU) will be considered failure. Stopping or switching due to dolutegravir/NRTI intolerance or AE will be regarded as failure. Switching for reasons of stopping contraception or wish to become pregnant, or becoming pregnant, transfer out for non-clinical reasons, and death from non-HIV and non-drug causes (as assessed by study investigators) will not be regarded as failure.

This study is not powered for formal statistical comparison between the two arms yet informal comparison of virologic suppression (point estimates and 95% CI) achieved in the two arms will be possible. There are precedents for conducting Phase II non-comparative trials with active controls arms to evaluate virologic suppression endpoints with InSTI-based ART regimens. The INSPIRING study randomised patients on tuberculosis treatment to dolutegravir or efavirenz-based treatment and described virologic suppression at 48 weeks with 95% CI without making claims on the statistical significance of comparisons ^23^.

The proportion achieving virologic suppression at week 24, stratified by NRTI resistance present at baseline, will be described. Genotypic resistance will be classified using the Stanford algorithm ^24^, with a score ≥15 indicating at least low-level resistance. Baseline NRTI resistance will be categorised as two fully active NRTIs (both with a Stanford score <15), resistance to one NRTI (one with a Stanford score <15 and one with a Stanford score ≥15) and dual resistance to both NRTIs (both with a Stanford score ≥15).

### Secondary endpoint analysis

A mITT analysis at weeks 12 and 48 will be performed to compare proportion achieving virologic suppression by study arm. The proportion achieving virologic suppression at weeks 12 and 48, stratified by NRTI resistance present at baseline, will be described. A secondary analysis will describe the proportion achieving VL <400 copies/mL at weeks 12, 24, and 48 by study arm using mITT analysis. A sensitivity analysis of virologic suppression at weeks 12, 24, and 48 will be performed, excluding certain participants included in the primary endpoint analysis: those LTFU or missing a VL in the window, those with evidence of poor adherence at the visit (TFV-DP concentrations <350 fmol/punch), and those changed from study medication for reasons other than treatment failure. Time-to-event endpoints will be analysed using the Kaplan-Meier survival analysis.

## Ethical considerations

### Confidentiality

All participant interactions will maintain strict confidentiality and names will be removed from the datasets for analysis. Participant identifiers (including study numbers linked to names) will be stored in one participant log file that will be locked in a cabinet. All other study documents will use the study number only.

### Consent

Informed consent will be obtained from every potential participant at screening and in advance of enrolment into the study. Participants will be assessed by the counsellor or study nurse taking consent for their capacity to consent. All potential participants providing consent will be ≥18 years old, with no clinical reason to suspect that they are not of sufficient capacity to consent. Consent will be explained verbally and in a written form. IsiXhosa speaking participants will have the option of a translated consent form in isiXhosa, with explanation by an isiXhosa-speaking healthcare worker. The study staff member taking consent will read through the informed consent form (ICF) ^25, 26^ with the participant and ensure that the participant understands the information provided in the ICF by asking the participant a set of comprehension questions about the trial.

The study staff member taking consent will complete a literacy assessment by asking the potential participant to read the first paragraph of the ICF and asking the participant if they can write their full name, the date, and their signature. In the case of an illiterate potential participant, a literate impartial adult witness will be present during each of the informed consent processes. All potential participants will be offered a copy of ICF clearly listing the risks and benefits of the trial.

### Consent for sample storage

A separate informed consent for genetic testing will be obtained from every participant at screening. All consenting participants will have an additional sample taken at enrolment. The samples will be stored for future genetic studies that are related to HIV and its treatment. Unwillingness to participate in genetic testing will not exclude patient from the main study.

### Non-maleficence

Intense monitoring of VL (at weeks 4, 8, 12, 16, 20, 24, 36, and 48) allows for a change in regimen if virologic suppression is not achieved or maintained. Virologic suppression will be monitored by DSMC who will convene every two months during the study to review virologic data with a pre-defined stopping rule based on failure to achieve a threshold of virologic suppression. If a participant has a VL >500 copies/mL despite enhanced adherence support, genotypic resistance testing will be performed, and the resistance testing results will be discussed with Trial Steering Committee to inform the design of a new suppressive regimen that could include standard of care protease inhibitors, darunavir, rilpivirine and/or raltegravir. We anticipate that such events, if they do occur, will be infrequent (around 5% of participants in dolutegravir monotherapy studies developed dolutegravir resistance ^10, 11^, and likely less with two NRTIs added).

Furthermore, we anticipate that because no participant will be protease inhibitor-experienced, a protease inhibitor-based alternative regimen will be sufficient in all cases where dolutegravir resistance develops. We anticipate that it is very unlikely such resistant viral mutants would result in onward transmission. The availability of suppressive alternative regimens makes this unlikely and the signature dolutegravir resistance mutation R263K severely compromises the virus’ replicative capacity, meaning that viruses carrying the mutation disappear rapidly from the circulating population of viruses when dolutegravir drug pressure is removed.

### Withdrawals

Participants reserve the right to withdraw from the study at any stage. The study team will try to ascertain the reason for the withdrawal. However, participants are under no obligation to provide reasons for their withdrawal. The participant will be encouraged (but will not be obliged) to complete a withdrawal study visit, which will either be an unscheduled visit or the next scheduled visit (whichever is more practical or convenient). At the withdrawal study visit a termination source document will be completed for the participant. All participants who withdraw from the study will be referred to their local clinic for on-going care.

### Ancillary and post-trial care

Participants will continue with the fixed dose combination of TLD after completion of the study (48 weeks). This treatment will be supplied by local community health clinics. The major long-term risk to participants is the emergence of antiretroviral resistance mutations and virologic failure. We will perform follow-up viral load monitoring at 72 and 96 weeks. Genotypic resistance testing will be done in participants with virologic failure, and any change in ART directed by the resistance testing results. Any compensation for trial related harm will be paid by the University of Cape Town’s indemnity insurance.

### Ethical approval

The trial has obtained approval from the Human Research Ethics Committee at the University of Cape Town (Ref: 039/2019). We will seek approval from the ethics committee for any future protocol amendments before implementation.

### Trial registration

The trial was registered on ClinicalTrials.gov under the registration number NCT03991013 (19 ^th^ June 2019).

## Trial committees

The trial sponsor is the University of Cape Town (UCT), South Africa. The trial management group (TMG) will oversee day-to-day management of the trial and is formed of the Principal Investigator, Lead and Co-investigators, Study Pharmacist, Data Systems Manager, Study Coordinator, and Clinical Projects Coordinator. The TMG will meet every two weeks. The Trial Steering Committee (TSC) has members of the TMG and two independent members. The TSC provides high level oversight and decision-making for the trial and advises on management of participants who develop virologic failure.

The DSMC comprises four independent members and is supported by the DSMC Statistician. The DSMC will convene via teleconference every two months to review cumulative study data to evaluate efficacy, safety, study conduct, scientific validity, and data integrity of the study. The DSMC will make recommendations regarding continuation, modification, or termination of any or all arms of the study. The DSMC is independent from the sponsor.

## Dissemination of results

Original articles arising from the trial will be submitted to peer-review journals with open access for publication. Authorship will be determined by substantial contributions to the conception or study design, data acquisition or analysis, drafting or revision of the manuscript, and final approval of the version to be published. We do not intend to use professional writers. We will also share results through abstract presentations at appropriate HIV scientific conferences. Anonymised individual patient data can be shared for further research upon written request to the Principal Investigator.

## Discussion

Two other ongoing trials are investigating recycling of tenofovir plus XTC backbone with dolutegravir in second-line. Both the Dolutegravir and Darunavir Evaluation in Adults Failing Therapy (D²EFT) study ^27^ and the Nucleosides and Darunavir/Dolutegravir In Africa (NADIA) study ^28^ are non-inferiority trials powered for formal comparisons between a regimen of dolutegravir with recycled tenofovir plus XTC and a regimen of dolutegravir with zidovudine plus lamivudine (NADIA) or a regimen of ritonavir-boosted darunavir with two NRTIs (D²EFT), and will have results available in 2021 or 2022. Neither study is using the lead-in supplementary dolutegravir dose strategy. In LMICs where patients with virologic failure on first-line ART were transitioned to TLD without a lead-in dose of dolutegravir, treatment-emergent dolutegravir resistance developed in a small number of patients who had virologic failure on the second-line TLD regimen ^29^. Our study results will address the key unanswered question of whether a lead-in dolutegravir dose is required to minimize the risk of developing dolutegravir resistance and treatment failure, in patients switching from an efavirenz-based regimen to TLD with a raised VL, in a Phase II design that could provide important data to supplement the findings of D²EFT and NADIA in informing policy or design of a Phase III trial.

## Study status

The study, with protocol version 4.1, 20 August 2020, began enrolment on 28 August 2020 and had recruited 45 participants by 17 January 2021. Estimated time for full accrual is one year.

## Data availability

### Underlying data

No data are associated with this article.

### Extended data

Figshare: ARTIST Informed Consent Form in English, https://doi.org/10.6084/m9.figshare.13681783.v1 ^25^.

Figshare: ARTIST Informed Consent Form in isiXhosa, https://doi.org/10.6084/m9.figshare.13681855.v1 ^26^.

### Reporting guidelines

Figshare: SPIRIT checklist for “AntiRetroviral Therapy In Second-line: investigating Tenofovir-lamivudine-dolutegravir (ARTIST): protocol for a randomised controlled trial”, https://doi.org/10.6084/m9.figshare.13681885.v1 ^30^.

Data are available under the terms of the Creative Commons Zero "No rights reserved" data waiver (CC0 1.0 Public domain dedication).
