# Supplementary material for: AntiRetroviral Therapy In Second-line: investigating Tenofovir-lamivudine-dolutegravir (ARTIST): protocol for a randomised controlled trial
Source: Wellcome Open Res. 2021 Feb 17;6:33. [Version 1] doi: 10.12688/wellcomeopenres.16597.1 (PMC9372637; doi:10.12688/wellcomeopenres.16597.1)
Supplement: Peer review history

# Peer review: Reviewer response for version 1

**DOI:** 10.21956/wellcomeopenres.18291.r42692

**Published:** 2022-08-11

**Authors:** Loice Achieng Ombajo

**Affiliations:** 1 Department of Clinical Medicine and Therapeutics, University of Nairobi, Nairobi, Kenya

Summary of the article:

 The article AntiRetroviral Therapy in Second-line, investigation Tenofovir-lamivudine-dolutegravir (ARTIST): protocol for a randomised controlled trial, aims to investigate whether a supplemental 50 mg dose of Dolutegravir given in addition to TLD in the initial two weeks of treatment (lead-in) reduces the risk of failure and emergence of DTG resistance in participants switching from a failing Tenofovir-emtricitabine-efavirenz regimen. This will be a randomised, double-blind clinical trial in which 130 participants will be randomised 1:1 to either TLD+DTG or TLD+matching placebo for the first two weeks; participants in each arm will subsequently continue with TLD. The investigators will assess the proportion of participants with VL<50 c/ml at week 24 in both arms using the FDA snapshot algorithm, secondary endpoints will include viral suppression at weeks 12 and 48, resistance to DTG in those failing TLD and its correlates, adherence using TDF levels and assessment of DTG trough levels. The investigators will also describe any adverse events. Ancillary studies will include pharmacokinetic studies in 24 participants and an assessment of genetic polymorphisms associated with DTG metabolism.

 This is a very well-written paper with a clear justification, it is relevant in contexts where TLE/TEE are still the predominant first-line agents. The pharmacokinetic questions are important. The methods, analysis, and data management sections are clear. The paper is suitable for indexing.

 I have a few comments and questions:

1. The first statement in the abstract as well as part of the literature review may need to be updated in view of the recent presentation of data from both the NADIA ^1^ and VISEND trials. The VISEND trial showed that TLD had superior efficacy to PI/r (LPVr and ATVr) and the NADIA showed that DTG did well even in the absence of a fully active NRTI.
2. In the inclusion criteria, I wonder if it would be clearer if written as: "Two consecutive viral loads >1000 copies/ml with the last taken within the previous 2 months."
3. Frequency of viral load vs assessment for adherence: It is not clear what role the week 8, 16, and 20 viral load measurements will serve, whereas week 4 may be an early safety marker to enable identification and response to any early failure, the other viral loads are not attached to any efficacy endpoint and this frequency of viral measurement does not give room for strengthening of adherence prior to the measurement of the next viral load.
4. What would the potential impact of switching to two pills in the initial two weeks (after the failure of a single pill regimen) as well as the high frequency of reviews be on adherence?
5. Increased DTG dose in the context of rifamycin use is clear. In the event that TB is diagnosed within the first two weeks, how will DTG be dosed in those patients already on the supplemental DTG arm?
6. How will missing data be handled? Where stopping/switching if for reasons other than failure, how will the last VL data be treated - by participant exclusion or as treatment success?

Is the study design appropriate for the research question?

Yes

Is the rationale for, and objectives of, the study clearly described?

Yes

Are sufficient details of the methods provided to allow replication by others?

Yes

Are the datasets clearly presented in a useable and accessible format?

Not applicable

Reviewer Expertise:

Infectious disease, HIV therapy, antimicrobial resistance

I confirm that I have read this submission and believe that I have an appropriate level of expertise to confirm that it is of an acceptable scientific standard.

## References

[1] : Efficacy and safety of dolutegravir or darunavir in combination with lamivudine plus either zidovudine or tenofovir for second-line treatment of HIV infection (NADIA): week 96 results from a prospective, multicentre, open-label, factorial, randomised, non-inferiority trial. *The Lancet HIV* .2022;9(6) : 10.1016/S2352-3018(22)00092-3 e381-e393 10.1016/S2352-3018(22)00092-3 35460601

# Peer review: Reviewer response for version 1

**DOI:** 10.21956/wellcomeopenres.18291.r51752

**Published:** 2022-08-11

**Authors:** Laura Waters

**Affiliations:** 1 Mortimer Market Centre, Central and North West London NHS Foundation Trust, London, UK

Optimising second-line ART in regions where viral load testing is limited, and resistance sequencing even more so, is an important research and clinical challenge. The rationale for the protocol is clear but I have the following concerns:

1. DAWNING is a key trial but has been somewhat superseded by NADIA, a similar trial using darunavir/ritonavir rather than lopinavir/ritonavir as the comparator. Since the 48 weeks results were published a year ago ^1^ and the 96-week results were presented this year, I am surprised that this major trial has not been referenced (bar a very brief mention and reference to ClinicaTrialsGov at the end). NADIA, by week 96, showed the superiority of recycled tenofovir-DF3TC vs switch to zidovudine/3TC so provides important corroborative support for this study's design.
2. NADIA has revealed a small but important risk of integrase resistance emergence amongst those experiencing virological failure on dolutegravir compared to no emergent protease resistance among those experiencing virological failure on darunavir/ritonavir - this should be acknowledged. What treatment will be offered to trial participants experiencing virological failure?
3. Have the authors analysed the timing and nature of virological failures in DAWNING, NADIA, and D2EFT? Certainly, for NADIA there were more virological failures between W48 and W96 than during the first year, so viral rebound is more of an issue than failure to suppress. If the same is true for similar trials, I am not sure how increasing the DTG dose early is helpful? If most VFs were driven by a failure to suppress it would make more sense?
4. As the authors point out, there remains a question as to whether DTG really is the optimal second-line option - especially as DTG is increasingly a first line option, will this whole study not be superseded by the most recent update to WHO guidelines? How relevant will the question be to the next generation of people experiencing first-line treatment failure? I believe the ideal trial design would be the two planned arms + a darunavir/ritoanvir arm, but I imagine this suggestion is beyond the scope of my review! These issues should at least be considered in the discussion which, I feel, is too brief.

Is the study design appropriate for the research question?

Partly

Is the rationale for, and objectives of, the study clearly described?

Partly

Are sufficient details of the methods provided to allow replication by others?

Yes

Are the datasets clearly presented in a useable and accessible format?

Not applicable

Reviewer Expertise:

HIV treatment; antiretroviral toxicites; HIV resistance

I confirm that I have read this submission and believe that I have an appropriate level of expertise to confirm that it is of an acceptable scientific standard, however I have significant reservations, as outlined above.

## References

[1] : Dolutegravir or Darunavir in Combination with Zidovudine or Tenofovir to Treat HIV. *New England Journal of Medicine* .2021;385(4) : 10.1056/NEJMoa2101609 330-341 10.1056/NEJMoa2101609 34289276

# Peer review: Reviewer response for version 1

**DOI:** 10.21956/wellcomeopenres.18291.r50587

**Published:** 2022-05-30

**Authors:** Stephen Ian Walimbwa

**Affiliations:** 1 Infectious Diseases Institute, College of Health Sciences, Makerere University, Kampala, Uganda

The use of a public health approach for the management of patients failing first-line ART suggests that DTG remains vulnerable to resistance, especially in patients with NRTI cross-resistance. In LMICs, access to resistance testing is limited; therefore, this study will provide evidence to support recommendations for DTG dosing in patients failing EFV-based first-line ART treatments during the immediate post-switch period.

- Indicate the CYP2B6 genetic polymorphisms associated with slow metabolizers of EFV within the study population.
- Table 1: For clarity, indicate the duration of the screening period (if any) and indicate the baseline visit column.
- Indicate the dissemination plan to participants.
- Update the discussion in the main text to highlight the findings from recently published data ^1^^,^^2^ on the use of DTG in 2 ^nd^ line ART in sub-Saharan Africa. These recent findings****** are relevant to the discussion of the protocol and also strengthen the rationale of the ARTIST protocol.

Is the study design appropriate for the research question?

Yes

Is the rationale for, and objectives of, the study clearly described?

Yes

Are sufficient details of the methods provided to allow replication by others?

Yes

Are the datasets clearly presented in a useable and accessible format?

Not applicable

Reviewer Expertise:

Pharmaceutical Medicine, Infectious Diseases, Pharmacokinetics, Immunopharmacology

I confirm that I have read this submission and believe that I have an appropriate level of expertise to confirm that it is of an acceptable scientific standard.

## References

[1] : Dolutegravir or Darunavir in Combination with Zidovudine or Tenofovir to Treat HIV. *New England Journal of Medicine* .2021;385(4) : 10.1056/NEJMoa2101609 330-341 10.1056/NEJMoa2101609 34289276

[2] : Efficacy and safety of dolutegravir or darunavir in combination with lamivudine plus either zidovudine or tenofovir for second-line treatment of HIV infection (NADIA): week 96 results from a prospective, multicentre, open-label, factorial, randomised, non-inferiority trial. *The Lancet HIV* .2022; 10.1016/S2352-3018(22)00092-3 10.1016/S2352-3018(22)00092-3 35460601
